# Comparison of IVF Outcomes between Minimal Stimulation and High-Dose Stimulation for Patients with Poor Ovarian Reserve

**DOI:** 10.1155/2014/581451

**Published:** 2014-10-01

**Authors:** Tal Lazer, Shir Dar, Ekaterina Shlush, Basheer S. Al Kudmani, Kevin Quach, Agata Sojecki, Karen Glass, Prati Sharma, Ari Baratz, Clifford L. Librach

**Affiliations:** ^1^CReATe Fertility Centre, 790 Bay Street, Suite 1100, Toronto, ON, Canada M5G 1N8; ^2^Department of Obstetrics & Gynecology, University of Toronto, Toronto, ON, Canada M5S 2J7; ^3^Division of Reproductive Endocrinology and Infertility, Department of Obstetrics and Gynecology, Women's College Hospital, Toronto, ON, Canada M5S 1B2

## Abstract

We examined whether treatment with minimum-dose stimulation (MS) protocol enhances clinical pregnancy rates compared to high-dose stimulation (HS) protocol. A retrospective cohort study was performed comparing IVF and pregnancy outcomes between MS and HS gonadotropin-antagonist protocol for patients with poor ovarian reserve (POR). Inclusion criteria included patients with an anti-Müllerian hormone (AMH) ≤8 pmol/L and/or antral follicle count (AFC) ≤5 on days 2-3 of the cycle. Patients from 2008 exclusively had a HS protocol treatment, while patients in 2010 had treatment with a MS protocol exclusively. The MS protocol involved letrozole at 2.5 mg over 5 days, starting from day 2, overlapping with gonadotropins, starting from the third day of letrozole at 150 units daily. GnRH antagonist was introduced once one or more follicles reached 14 mm or larger. The HS group received gonadotropins (≥300 IU/day) throughout their antagonist cycle. Clinical pregnancy rate was significantly higher in the MS protocol compared to the HS protocol (*P* = 0.007). Furthermore, the live birth rate was significantly higher in the MS group compare to the HS group (*P* = 0.034). In conclusion, the MS IVF protocol is less expensive (lower gonadotropin dosage) and resulted in a higher clinical pregnancy rate and live birth rate than a HS protocol for poor responders.

## 1. Introduction

Patients with poor ovarian response (POR) are both challenging to treat and represent a large proportion of patients presenting with infertility [1, 2]. Patients with POR, who are often of advanced maternal age, have a high cycle cancellation rate, higher miscarriage rate, and significantly reduced live birth rate per cycle. To date, there is no universally accepted definition for POR. These patients generally have one or more of the following characteristics: advanced maternal age, low AMH levels, high FSH in the early follicular phase (~day 3) (≥10 mIU/mL), low early follicular phase antral follicle count (AFC) (3–7) [3, 4], low number of mature retrieved oocytes (<4) after superovulation with a moderate to high-dose protocol, low peak E2 levels (<3300 pmol/L), and prior cycle cancellation(s) due to poor response [5–7]. The European Society of Human Reproduction and Embryology (ESHRE) attempted to standardize the definition of POR in 2010 and this resulted in a consensus definition called the Bologna criteria. At least two of the following three features must be present: (1) advanced maternal age (≥40 years) or any other risk factors for POR, (2) a previous POR (≤3 oocytes) with a conventional stimulation protocol, and/or (3) an abnormal ovarian reserve test (AFC < 5–7 follicles or AMH < 0.5–1.1 ng/mL) (REF).

The management of POR is highly controversial as well. There is still no consensus regarding the “ideal” protocol and so far no one treatment protocol has proven to be superior for this group. The majority of the strategies aim to recruit a higher number of follicles either by increasing the dose of gonadotropins, decreasing the dose of GnRH analogs, suppressing an early rise in FSH with “estrogen priming,” or optimizing the endogenous FSH flare effect [1]. In addition, adjunctive growth hormone is advocated by some studies [1, 7] while aromatase inhibitors have also been suggested in other studies [8].

Letrozole is a potent and highly specific nonsteroidal third generation aromatase inhibitor, originally approved for use in postmenopausal women with hormone receptor positive breast cancer to suppress estrogen production [9]. It inhibits the aromatase enzyme resulting in decreased estradiol synthesis. Letrozole is increasingly being utilized for ovulation induction in infertility. By decreasing early follicular phase estrogen synthesis, there is a decrease in estradiol-mediated negative feedback at the hypothalamus, with a resultant increase in endogenous gonadotropin secretion. Healey et al. [10] demonstrated that the addition of letrozole to gonadotropins increases the number of preovulatory follicles without having a negative impact on pregnancy rate. In addition, letrozole was found to cause an increase in intraovarian androgen levels which in turn increases FSH receptor expression on follicular granulosa cells [11]. Thus, letrozole may improve the ovarian response to FSH in poor responders [11].

In our study, we compared a standard high-dose gonadotropin-antagonist (HS) protocol for poor responders to a minimal stimulation (MS) protocol involving letrozole overlapping with a low dose of gonadotropins, for poor responders. Our hypothesis was that using a MS protocol with letrozole might enhance clinical pregnancy rates over a HS protocol.

## 2. Materials and Methods

### 2.1. Patients

This is a retrospective cohort study using data from IVF cycles in patients with poor ovarian reserve, carried out at the CReATe Fertility Centre in Toronto, Canada. The inclusion criteria included patients with poor ovarian reserve as defined by the Bologna criteria [12]. Due to predicted poor outcome, cycles where only a single dominant follicle developed were cancelled and excluded.

In 2008 we were performing exclusively HS protocols on these patients. There were a total of 71 IVF cycles that met these criteria during that year. During 2009, after some early positive reports on MS, we began using a MS protocol in an attempt to improve pregnancy rates in this poor prognosis population. Based on our early observations of higher success with this protocol, by 2010 we transitioned to using, almost exclusively, the MS protocol for this group of patients. There were a total of 70 cycles that met these criteria during 2010. Therefore, we compared our 2008 cycles to 2010 in order to avoid selection bias. All cycles included for each group represented their first IVF attempt. This study was approved by the University of Toronto Research Ethics Committee (REB Approval number 28824).

### 2.2. Treatment Protocol

**Figure 1:**
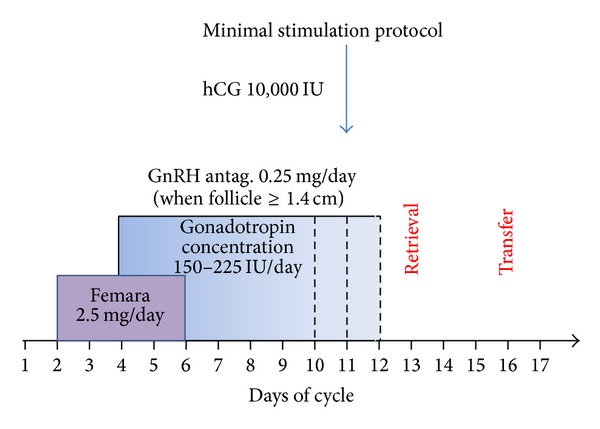
Treatment scheme for the minimal stimulation protocol.

All patients had an initial transvaginal ultrasound examination to measure the uterine lining and perform an antral follicle count on day 2 of the cycle. Baseline blood levels of estradiol, FSH, LH, and progesterone were also measured at the same visit. The MS protocol consisted of low dose letrozole (Femara; Novartis, Dorval, Quebec, Canada) 2.5 mg PO over 5 days, starting from cycle day 2 (Figure 1). On day 4 of the cycle (day 3 of the letrozole treatment) overlapping low dose gonadotropins, menopur (Ferring Inc., Toronto, ON, Canada) was initiated. The initial gonadotropin dose was 150 IU per day. After 3 days on menopur, the patient was reviewed for standard ultrasound and blood hormone monitoring and the dose was titrated according to the initial response. Depending on the response, the gonadotropin dose was either maintained or increased up to 225 IU, but not higher. When one or more follicles reached 14 mm in size, gonadotropin releasing hormone (GnRH) antagonist, cetrotide (EMD Serono, Darmstadt, Germany) 0.25 mg, was introduced to avoid a premature LH surge. Human chorionic gonadotropin (hCG) (PPC, Richmond Hill, ON, Canada) 10000 IU was administered for final maturation when at least 1 or 2 follicles reached 18 mm or above. Cycles where there was a single dominant follicle were cancelled. Oocyte retrieval was performed approximately 36 hours after the hCG injection. Intracytoplasmic sperm injection (ICSI) was performed in almost all cases in order to optimize fertilization for the small number of oocytes. The control group received high levels of gonadotropins (≥300 IU/day) starting from day 2 of their cycle and throughout their short antagonist cycle. Antagonist initiation, hCG administration timing, and oocyte retrieval timing were the same as those for the MS protocol.

Embryo transfer was performed on day 3 under ultrasound guidance. Luteal support was with either intramuscular administration progesterone in ethyl oleate oil (2 cc of 50 mg/mL) (compounded by our local pharmacist (R.B.)) or progesterone suppositories 100 mg qid (compounded by our local pharmacist (R.B.)), started on the day of retrieval. Serum *β*hCG levels were tested starting 2 weeks after embryo transfer, and then serially, if positive, followed by a transvaginal ultrasound examination between 6 and 7 weeks of gestation. Clinical pregnancy was defined for this study as the presence of a gestational sac, with or without fetal cardiac activity.

Data were expressed as mean ± standard deviation. The student's *t*-test and *χ* ^2^ testing were used for data comparisons adjusting variables using a 95% confidence interval. A *P* value less than 0.05 was considered statistically significant. SPSS version 15.0 (SPSS Inc. Chicago, IL, USA) was used for data analysis.

## 3. Results

A total of 141 cycles (*n* = 70 for 2010 and *n* = 71 for 2008) met the inclusion criteria. Patients' demographic and clinical data are shown in Table 1. There was no significant difference between the MS and HS groups with respect to age (39.1 ± 3.8 versus 39.0 ± 3.9, resp.). The E2 level on the day of hCG administration (MS 1580.8 ± 1141.2 versus HS 5575 ± 3295.1 pmol/L, *P* < 0.001) and the total units gonadotropins administered during the stimulation protocol (MS 1332.9 ± 435.7 versus HS 5575.2 ± 1945 IU, *P* < 0.001) were significantly higher in the HS group. There was no significant difference in the number of oocytes retrieved with the MS versus HS protocol (2.9 ± 1.5 versus 3.5 ± 1.5 resp., *P* > 0.05). Cancelled cycles due to the formation of a single dominant follicle represented 5% of cycles started in each group (NS). There was no significant difference in the number of fertilized eggs (2PN) between the two protocols (1.5 ± 1.1 versus 1.5 ± 1.2 resp., *P* > 0.05), and no significant difference in the number of embryos transferred per cycle for the MS versus the HS protocols (1.8 ± 0.7 versus 1.4 ± 1.2 resp., *P* > 0.05) was found. Clinical pregnancy rate was significantly higher in the MS versus HS protocols (22/70, 31.4% versus 9/78, 12.7%, resp., *P* < 0.05). The live birth rate was significantly higher in the MS group compared to the HS group (15/70, 21.4% versus 5/71 7.0%, resp., *P* < 0.05).

A power analysis was conducted for a chi-square of the two groups. We assumed a significance level of 0.05 and a power level of 0.80. At an “*n*” of 79 for group 1 (2008) and a proportion of 0.14, the largest and smallest detectable proportions for group 2 (2010) are 0.35 and 0.0097, respectively.

## 4. Discussion

Treating patients with poor ovarian response remains one of the biggest challenges in reproductive medicine. It is usually a problem of advanced reproductive age patients [13]; however previous ovarian surgery [14], pelvic infections [15], and environmental factors or genetic factors may be associated with it in younger patients as well [16]. The objective of this study was to compare IVF laboratory results, clinical pregnancy rate, and live birth rate from a MS protocol using a combination of letrozole (aromatase inhibitor) and low dose gonadotropins versus a HS protocol. We found that our MS protocol resulted in a significantly higher clinical pregnancy rate and live birth rate.

There is no consistent definition for a “poor responder” in the literature and so it is difficult to compare studies and reach consensus on treatment effects. The ESHRE consensus, otherwise known as the Bologna criteria, establishes a guideline for poor ovarian reserve. However, this guideline is not universally accepted. Poor responders often present with a shortened follicular phase, which decreases the time available for follicular recruitment. In addition, lower FSH receptor expression levels in granulosa cells may also be found in this group of patients [17]. In order to overcome the shorter follicular phase and create a longer window of opportunity for follicular recruitment, we hypothesized that the introduction of an aromatase inhibitor may be beneficial, through decreasing estrogen levels and prolonging the action of FSH. In addition, a letrozole-mediated increase in intraovarian androgen concentration may improve ovarian responsiveness to exogenous gonadotropins in poor responders. Hillier [18] was the first to introduce the idea of androgens as a treatment to promote gonadotropin responsiveness in granulosa cells. Weil et al. [19] suggested that androgen treatment may promote follicular growth and estrogen biosynthesis. Androgens are also known to stimulate IGF-1 and IGF-1 receptor gene expression which are known to promote follicular steroidogenesis [1, 17].

Letrozole is a potent, low cost, and highly specific nonsteroidal aromatase inhibitor administered orally. It inhibits the aromatase enzyme by competitively binding to the heme domain of the enzyme's cytochrome P450 subunit, resulting in a blockade of androgen conversion into estrogens with a subsequent increase in intraovarian androgens [17]. Garcia-Velasco et al. [17] in a prospective pilot study were able to demonstrate that aromatase inhibition with letrozole at the beginning of ovarian stimulation significantly increases intraovarian androgen concentrations. They showed that follicular fluid levels of testosterone and androstenedione are significantly elevated in women given letrozole during ovarian stimulation for IVF. Other investigators have advocated the use of androgen supplementation, such as DHEA [20], but this is controversial. It is hypothesized that androgens or aromatase inhibitors may play a role in preantral and antral follicular development and may therefore improve ovarian responsiveness.

MS usually refers to stimulation protocols that yield a maximum of five oocytes [5]. This concept was first introduced by Corfman et al. [21]. Their MS protocol involved using CC 100 mg orally on days 3–7 of the cycle, followed by a single injection of 150 IU of IM hMG on cycle day 9. Although the number of retrieved oocytes was statistically lower using a MS protocol, this variability did not correlate to a statistically significant difference in pregnancy rate [21]. Subsequently, many papers introduced a variety of different protocols termed “minimal stimulation,” which combined CC or letrozole with low levels of gonadotropins.

Although there is no conclusive data regarding the optimal doses for letrozole in reproductive medicine, letrozole at doses of 1–5 mg/day inhibits aromatase activity by 97–99% [11, 17]. Most published studies have involved a once-daily dose of 2.5–5 mg for 5 days [22]. A randomized study comparing 2.5 and 5 mg of letrozole in women with unexplained infertility suggested that a higher dose of letrozole might be associated with the development of more follicles in patients with normal ovarian reserve. However, higher doses of letrozole were found to cause persistent inhibition of aromatase and lower estrogen levels for endometrial growth at ovulation [11]. In our protocol, we chose to use the lower dose of letrozole (2.5 mg), to avoid oversuppression and it was our group's clinical impression that this dose was more effective for patients with poor ovarian reserve. However, a RCT for the poor responder population would be required to test this hypothesis.

Conversely, we hypothesize that higher levels of gonadotropins, as well as the resultant very high levels of estrogen, may negatively impact the growing follicles, oocytes, and the endometrium, thus reducing the chances of a successful pregnancy for poor responders.

Mohsen and El Din [23] showed no difference in clinical pregnancy rates in poor responders when comparing a GnRHa microflare protocol to a letrozole antagonist protocol; however the days of stimulation and total dose of gonadotropins were significantly lower in the letrozole group. Yarali et al. [9] also studied a microdose GnRH agonist protocol versus a letrozole antagonist protocol in poor responders. They used a lower dose of gonadotropins and there was a shorter duration of treatment for the letrozole antagonist group, but again there was no difference in pregnancy rate. As a result, the minimal stimulation protocol tended to be easier to tolerate for patients and the cost of medication was greatly reduced.

Strengths: our fertility practices changed during the two years of the study from using one stimulation protocol (HS) to using another stimulation (MS) protocol in the poor ovarian reserve, poor responder population. The HS and MS protocols were used exclusively in 2008 and 2010, respectively. Thus, there was no bias towards choosing one protocol over the other in studied time periods.

Weaknesses: since we were comparing two groups, from two years apart (2008 and 2010), it is possible that the increase in pregnancy rate we observed was due to unrelated improvements in our embryology laboratory between these two years. To address this possibility, we compared pregnancy rates in normal and high responders and in egg donation cycles between these two years and there were no significant differences. In our clinic, egg donors had a pregnancy success rate of 53.6% and 52.5% in 2008 and 2010, respectively (*P* > 0.05). Normal responders of all ages had an overall pregnancy success rate of 35.3% and 32.8% in 2008 and 2010, respectively (*P* > 0.05). In 2008 and 2010, high responders had a pregnancy success rate of 41.4% and 38.6%, respectively (*P* < 0.05). Since one protocol being compared involved letrozole and the other did not, we cannot be certain that our results were not related to the use of letrozole rather than high versus low dose gonadotropins. Since this is a retrospective cohort study, in order to address this issue and confirm our overall findings, an adequately powered, prospective randomized controlled trial would be required. In addition, to determine if our findings are due to the lower dose of gonadotropins or the use of letrozole in this protocol, one would require a 3-armed prospective trial.

In conclusion, the MS protocol was less expensive (in terms of total gonadotropins used) and improved both clinical pregnancy and live birth rates, compared to the HS protocol. Based on our results, a prospective randomized controlled trial is warranted in order to confirm these findings.

## Figures and Tables

**Table 1:** Data comparison of minimal stimulation and high-dose stimulation protocols for low responders. Not significant results are denoted by NS.

	Minimum stimulation	High stimulation	*P* value
Number of patients	70	71	
Age (yr)	39.4 ± 3.2	39.2 ± 4.0	NS
Peak estradiol (pmol/L)	1580.8 ± 1141.2	5279.4 ± 3295.1	*P* < 0.001
Gonadotropin total dose (IU)	1332.9 ± 435.7	5575.2 ± 1945.0	*P* < 0.001
Antral follicle count	3.7 ± 1.0	4.5 ± 0.7	*P* < 0.001
Number of oocytes retrieved	2.9 ± 1.5	3.5 ± 1.5	NS
Number of fertilized oocytes	1.5 ± 1.1	1.5 ± 1.2	NS
Cancellation rate	3/71 (4.2%)	4/79 (5.0%)	NS
Number of embryos transferred	1.8 ± 0.7	1.4 ± 1.2	NS
Clinical pregnancy rate/cycle	22/70 (31.4%)	9/71 (12.7%)	*P* < 0.05
Live birth rate	15/70 (21.4%)	5/71 (7.0%)	*P* < 0.05
